# Core concepts of human rights and inclusion of vulnerable groups in the mental health policies of Malawi, Namibia, and Sudan

**DOI:** 10.1186/1752-4458-7-7

**Published:** 2013-02-13

**Authors:** Hasheem Mannan, Shahla ElTayeb, Malcolm MacLachlan, Mutamad Amin, Joanne McVeigh, Alister Munthali, Gert Van Rooy

**Affiliations:** 1 Centre for Global Health & School of Psychology, Trinity College Dublin, Dublin, Ireland; 2 School of Psychology at Ahfad University for Women, Omdurman, Sudan; 3 Centre for Global Health & School of Psychology, Trinity College Dublin, Dublin, Ireland, and Centre for Rehabilitation Studies, Stellenbosch University, Stellenbosch, South Africa; 4 Ahfad University for Women, Omdurman, Sudan; 5 Centre for Social Research, University of Malawi, Zomba, Malawi; 6 Multidisciplinary Research Centre, University of Namibia, Windhoek, Namibia

**Keywords:** Mental health policy, Core concepts of human rights, Vulnerable groups, Malawi, Namibia, Sudan

## Abstract

**Background:**

One of the most crucial steps towards delivering judicious and comprehensive mental health care is the formulation of a policy and plan that will navigate mental health systems. For policy-makers, the challenges of a high-quality mental health system are considerable: the provision of mental health services to all who need them, in an equitable way, in a mode that promotes human rights and health outcomes.

**Method:**

*EquiFrame*, a novel policy analysis framework, was used to evaluate the mental health policies of Malawi, Namibia, and Sudan. The health policies were assessed in terms of their coverage of 21 predefined Core Concepts of human rights (Core Concept Coverage), their stated quality of commitment to said Core Concepts (Core Concept Quality), and their inclusion of 12 Vulnerable Groups (Vulnerable Group Coverage). In relation to these summary indices, each policy was also assigned an Overall Summary Ranking, in terms of it being of *High*, *Moderate*, or *Low* quality.

**Results:**

Substantial variability was identified across *EquiFrame’s* summary indices for the mental health policies of Malawi, Namibia, and Sudan. However, all three mental health policies scored high on Core Concept Coverage. Particularly noteworthy was the Sudanese policy, which scored 86% on Core Concept Coverage, and 92% on Vulnerable Group Coverage. Particular deficits were evident in the Malawian mental health policy, which scored 33% on Vulnerable Group Coverage and 47% on Core Concept Quality, and was assigned an Overall Summary Ranking of *Low* accordingly. The Overall Summary Ranking for the Namibian Mental Health Policy was *High*; for the Sudanese Mental Health Policy was *Moderate*; and for the Malawian Mental Health Policy was *Low*.

**Conclusions:**

If human rights and equity underpin policy formation, it is more likely that they will be inculcated in health service delivery. *EquiFrame* may provide a novel and valuable tool for mental health policy analysis in relation to core concepts of human rights and inclusion of vulnerable groups, a key practical step in the successful realization of the Millennium Development Goals.

## Background

Delivering judicious and comprehensive mental health care requires, as a critical step, the formulation of a policy and plan that will direct mental health systems and services development [1]. While imperative for improving conditions for persons with mental disabilities, mental health policies are either absent or deficient in the majority of countries of the world however [2]. Approximately forty percent of countries do not have a dedicated mental health policy [3]. The scale and cost of mental health difficulties indicate that appropriate policies must be developed and implemented [4]. Mental health policies define the vision for the future mental health of a population, specifying the framework that will be established to manage and prevent priority mental and neurological concerns; when clearly conceptualized, a mental health policy can coordinate critical services to safeguard the delivery of healthcare to those in need while concurrently preventing fragmentation and inefficiencies in the health system [5]. For policy-makers, the challenges of a high-quality mental health system are considerable: the provision of mental health services to all who need them, in an equitable way, adopting an approach that is highly effective, and in a mode that promotes human rights and health outcomes [1].

Policies need to specifically promote the human rights of people with mental disabilities in the actions that are there within prescribed; this obligation is founded on international human rights standards [6]. For example, the United Nations Convention on the Rights of Persons with Disabilities [7] stipulates that ‘States Parties undertake: To take into account the protection and promotion of the human rights of persons with disabilities in all policies and programmes’ (art 4). Throughout the world people with mental disabilities confront an extensive range of human rights violations, including lack of access to basic mental health care and treatment and the absence of community based mental health care, equating to a global human rights emergency in mental health [2]. Human rights, including the rights to non-discrimination, privacy, autonomy, and participation, are imperative in the design, implementation, monitoring and evaluation of mental health policies [8].

It is important to consider that while poor mental health can be a cause of the experience of social, civil, political, economic, and environmental inequalities, mental health difficulties can also be a consequence of such inequalities [9]. Current research reveals that mental health concerns are more prevalent among certain social groups than others, in some measure due to socio-economic and cultural position in society; for example depression is more common among women, and there are higher rates of certain mental health conditions among ethnic minorities [10]. Mental health conditions demonstrate a high prevalence among people living in poverty, people with HIV/AIDS, people in emergency settings, and other vulnerable groups [9]. Stigmatization and marginalization of vulnerable groups may result in isolation, a significant risk factor for future mental health difficulties, and may generate poor self-esteem, diminished motivation, and less hope for the future [9], and may undermine self-confidence and therefore identity itself, thereby strongly influencing our internal scripts for who we are and what we can do [11].

Mental health has been posited as one of the most neglected yet critical development issues in the realization of the Millennium Development Goals [12-14]. The Movement for Global Mental Health [15-17] has recently emerged in response to the Call for Action [18] for the scaling up of coverage of services for mental disorders globally. The Movement aims to improve the availability, accessibility, and quality of services for people with mental disorders worldwide, but particularly in low- and middle-income countries where effective services are frequently scarce, through the scaling up of services based on scientific evidence and human rights [19].

*EquiFrame* is a novel analytical and peer-reviewed framework that serves to identify the strengths and weaknesses in current health policy according to the degree to which a policy promotes and protects core concepts of human rights in healthcare, particularly among vulnerable groups. *EquiFrame* evaluates the degree of explicit commitment of an existing health policy to 21 core concepts of human rights and to 12 vulnerable groups, guided by the ethos of *universal*, *equitable* and *accessible* health services. Health policies established on the values of equity are more likely to result in health services that are more justly distributed within the population. This requires that policy-makers strive to ensure the provision of health services for all, particularly for vulnerable groups, in response to their needs [20]. The capacity of technological interventions alone to address health inequities is increasingly disputed; realization of the right to health requires intrepid political leadership that addresses the health needs of vulnerable groups, while promoting gender equality, and establishing effective, functioning health systems [21]. If human rights and equity underpin policy formation, it is more likely that they will be inculcated in health service delivery.

While the development and process of the framework has been described in greater detail elsewhere [22-24], this paper details the findings of the application of *EquiFrame* in relation to the existing mental health policies of Malawi, Namibia, and Sudan. We sought to identify, at the policy level, the degree of commitment of the Malawian, Namibian, and Sudanese mental health policies to core concepts of human rights and their inclusion of vulnerable groups. By so doing, our goal was to distinguish best-practice mental health policies, and to identify policies that may necessitate urgent revision.

## Method

### Development of *EquiFrame*

There is paucity of literature that outlines and utilizes analytical frameworks for the content of policies, or policy ‘on the books’ [25]. There is however a body of research on the process of health policy development [26,27]. While this body of research focuses on the critical importance of how policy is made, only little guidance is offered on evaluating policy ‘on the books’. Developing and applying a method for analyzing the actual content of policies was the focus of the present research. *EquiFrame* has been devised with the intention of developing a health policy analysis framework that would be of particular relevance in low-income countries in general, and in Africa in particular, and is guided by the ethos of *universal*, *equitable*, and *accessible* health services. *EquiFrame* has been developed as part of a Work Package led by Ahfad University for Women, Sudan, within a larger EU FP7 funded project, EquitAble, which is led by the Centre for Global Health at Trinity College Dublin, and which has a consortium of international partners (see http://www.equitableproject.org).

### Selection of policies

\The World Health Report, ‘Working Together for Health’ [28], noted that Africa has the greatest disease burden of any continent but has the poorest health services. The three African countries that are the focus of this analysis each represent distinct challenges in terms of equitable access to healthcare. These three countries allow us to address how access to the healthcare systems for vulnerable groups can best be promoted in contexts where a large proportion of the population has been displaced (Sudan); where the population is highly dispersed (Namibia); and where chronic poverty and high disease burden compete for meagre resources (Malawi).

*EquiFrame* has been applied in the analysis of 51 health policies across Malawi, Namibia, Sudan, and South Africa. Health policies were included if they met the following criteria: (1) Health policy documents produced by the Ministry of Health; (2) Policies addressing health issues outside of the Ministry of Health; (3) Strategies that address health policies; and (4) Policies related to the top 10 health conditions identified by the World Health Organization within the respective country. A search was carried out to locate available health policies. The relevant ministries, agencies, and libraries were contacted and asked to identify policy documents falling within the scope of our research. In this paper, we present our analysis of the mental health policies of Malawi, Namibia, and Sudan. We sought to assess the extent to which the mental health policies of these three countries promoted universal, equitable, and accessible health service provision.

### The framework

‘Core Concept’ may be defined as a “central, often foundational policy component generalized from particular instances (namely, literature reviews, analyses of statutes and judicial opinions, and data from focus groups and interviews)” [29]. *EquiFrame’s* 21 Core Concepts are presented alongside series of key questions and key language, each series tailored to elucidate the specified Core Concept (see Table 1). These 21 Core Concepts represent a broad range of salient concerns in striving for universal, equitable and accessible healthcare.

**Table 1:** ***EquiFrame *core concepts key questions and key language**

**No**	**Core concept**	**Key question**	**Key language**
1.	**Non-discrimination**	Does the policy support the rights of vulnerable groups with equal opportunity in receiving health care?	Vulnerable groups are not discriminated against on the basis of their distinguishing characteristics (i.e. Living away from services; Persons with disabilities; Ethnic minority or Aged).
2.	**Individualized Services**	Does the policy support the rights of vulnerable groups with individually tailored services to meet their needs and choices?	Vulnerable groups receive appropriate, effective, and understandable services.
3	**Entitlement**	Does the policy indicate how vulnerable groups may qualify for specific benefits relevant to them?	People with limited resources are entitled to some services free of charge or persons with disabilities may be entitled to respite grant.
4	**Capability based Services**	Does the policy recognize the capabilities existing within vulnerable groups?	For instance, peer to peer support among women headed households or shared cultural values among ethnic minorities.
5.	**Participation**	Does the policy support the right of vulnerable groups to participate in the decisions that affect their lives and enhance their empowerment?	Vulnerable groups can exercise choices and influence decisions affecting their life. Such consultation may include planning, development, implementation, and evaluation.
6.	**Coordination of Services**	Does the policy support assistance of vulnerable groups in accessing services from within a single provider system (interagency) or more than one provider system (intra-agency) or more than one sector (inter-sectoral)?	Vulnerable groups know how services should interact where inter-agency, intra-agency, and inter-sectoral collaboration is required.
7.	**Protection from Harm**	Are vulnerable groups protected from harm during their interaction with health and related systems?	Vulnerable group are protected from harm during their interaction with health and related systems.
8	**Liberty**	Does the policy support the right of vulnerable groups to be free from unwarranted physical or other confinement?	Vulnerable groups are protected from unwarranted physical or other confinement while in the custody of the service system/provider.
9.	**Autonomy**	Does the policy support the right of vulnerable groups to consent, refuse to consent, withdraw consent, or otherwise control or exercise choice or control over what happens to him or her?	Vulnerable groups can express “independence” or “self-determination”. For instance, person with an intellectual disability will have recourse to an independent third party regarding issues of consent and choice.
10.	**Privacy**	Does the policy address the need for information regarding vulnerable groups to be kept private and confidential?	Information regarding vulnerable groups need not be shared among others.
11.	**Integration**	Does the policy promote the use of mainstream services by vulnerable groups?	Vulnerable groups are not barred from participation in services that are provided for general population.
12.	**Contribution**	Does the policy recognize that vulnerable groups can be productive contributors to society?	Vulnerable groups make a meaningful contribution to society.
13.	**Family Resource**	Does the policy recognize the value of the family members of vulnerable groups in addressing health needs?	The policy recognizes the value of family members of vulnerable groups as a resource for addressing health needs.
14.	**Family Support**	Does the policy recognize individual members of vulnerable groups may have an impact on the family members requiring additional support from health services?	Persons with chronic illness may have mental health effects on other family members, such that these family members themselves require support.
15.	**Cultural Responsiveness**	Does the policy ensure that services respond to the beliefs, values, gender, interpersonal styles, attitudes, cultural, ethnic, or linguistic, aspects of the person?	i) Vulnerable groups are consulted on the acceptability of the service provided.
			ii) Health facilities, goods and services must be respectful of ethical principles and culturally appropriate, i.e. respectful of the culture of vulnerable groups.
16.	**Accountability**	Does the policy specify to whom, and for what, services providers are accountable?	Vulnerable groups have access to internal and independent professional evaluation or procedural safe guard.
17.	**Prevention**	Does the policy support vulnerable groups in seeking primary, secondary, and tertiary prevention of health conditions?	
18.	**Capacity Building**	Does the policy support the capacity building of health workers and of the system that they work in addressing health needs of vulnerable groups?	
19.	**Access**	Does the policy support vulnerable groups-physical, economic, and information access to health services?	Vulnerable groups have accessible health facilities (i.e., transportation; physical structure of the facilities; affordability and understandable information in appropriate format).
20.	**Quality**	Does the policy support quality services to vulnerable groups through highlighting the need for evidence-based and professionally skilled practice?	Vulnerable groups are assured of the quality of the clinically appropriate services.
21.	**Efficiency**	Does the policy support efficiency by providing a structured way of matching health system resources with service demands in addressing health needs of vulnerable groups?	

Vulnerable Groups may be defined as “social groups who experience limited resources and consequent high relative risk for morbidity and premature mortality” [30], and this may include children, the aged, ethnic minorities, displaced populations, people suffering from chronic illnesses and persons with disabilities. Rights approaches that prioritize those who are most vulnerable inherently promote equity by privileging those who are most marginalized [31]. The above definition of vulnerable groups resonates with the idea that vulnerability should be related to claims for special protection (for instance, in health policies), where there is a) a greater likelihood of people experiencing “wrongs”, and b) a duty to avoid identifiable “wrongs” [32]. Importantly, Eichler and Burke [33] have recognized that the social discrimination and bias that arises based on such categories is the result of social hierarchies: similar exclusionary practices disadvantage and disempower different groups, undermining their human rights and their rights to health, other social services and to social inclusion – to being full participants in society.

The World Report on Disability [34] estimates that over one billion people, or approximately 15% of the world’s population, are living with disability; yet many people with disabilities do not have equal access to health care, education, and employment opportunities, do not receive the disability-related services that they need, and encounter exclusion from everyday activities [34]. Accordingly, a particular interest of the research team was to assess the degree to which persons with disabilities (identified by *EquiFrame* as a Vulnerable Group) were incorporated in policy documents for the purpose of promoting more accessible healthcare.

To draw up a comprehensive list of appropriate social groups, we conducted a literature review spanning the international and national literatures. The resulting list was then refined and integrated to produce a categorization that would be credible for the broader analysis of health policies across the four project countries, as well as regional and international health policies. However, it was evident that there was also a need for flexibility for the purpose of accommodating any additional country-specific groups, where integration of them into another theme might miss the opportunity to provide valuable information. Vulnerable Groups outlined by *EquiFrame* are provided in Table 2, and these resonate with the “Social Determinants Approaches to Public Health” report [35].

**Table 2:** ***EquiFrame *vulnerable groups definitions**

**No.**	**Vulnerable group**	**Attributes or Definitions**
1.	**Limited Resources**	Referring to poor people or people living in poverty
2.	**Increased Relative Risk For Morbidity**	Referring to people with one of the top 10 illnesses, identified by WHO, as occurring within the relevant country
3.	**Mother Child Mortality**	Referring to factors affecting maternal and child health (0-5 years)
4.	**Women Headed Household**	Referring to households headed by a woman
5.	**Children (with special needs)**	Referring to children marginalized by special contexts, such as orphans or street children
6.	**Aged**	Referring to older age
7	**Youth**	Referring to younger age without identifying gender
8.	**Ethnic Minorities**	Referring to non-majority groups in terms of culture, race or ethnic identity
9.	**Displaced Populations**	Referring to people who, because of civil unrest or unsustainable livelihoods, have been displaced from their previous residence
10.	**Living Away from Services**	Referring to people living far from health services, either in time or distance
11.	**Suffering from Chronic Illness**	Referring to people who have an illness which requires continuing need for care
12.	**Disabled**	Referring to persons with disabilities, including physical, sensory, intellectual or mental health conditions, and including synonyms of disability

*EquiFrame* has been devised with the aim of generating a systematic evaluative and comparative analysis of health policies on technical content and design. In its current form, *EquiFrame* is directed towards health policy-oriented researchers and policy-makers. The Framework has been presented at a workshop conducted for the Ministry of Health in Malawi comprising senior policy-makers, and has provided guidance towards the redrafting of the Malawian National Health Policy. We believe therefore that the utility of *EquiFrame* will extend beyond a tool for evaluation of policies to the promotion of equity, human rights and inclusion in the revision of existing policies and development of new policies. For further details specific to *EquiFrame* and the process of its formulation, including a discussion of literature sources for Core Concepts and Vulnerable groups, readers are referred to the *EquiFrame* manual [22] (see also [23,24,36-41]).

### Summary indices

The four summary indices of *EquiFrame* are outlined below:

(1) **Core Concept Coverage**: A policy was examined with respect to the number of Core Concepts mentioned of the 21 Core Concepts identified; and this ratio was expressed as a rounded up percentage. In addition, the actual terminologies used to explain the Core Concepts within each document were extracted to allow for future qualitative analysis and cross-checking between raters [22-24; 36-41].

(2) **Vulnerable Group Coverage**: A policy was examined with respect to the number of Vulnerable Groups mentioned of the 12 Vulnerable Groups identified; and this ratio was expressed as a rounded up percentage. In addition, the actual terminologies used to describe the Vulnerable Groups were extracted to allow for qualitative analysis and cross-checking between raters.

(3) **Core Concept Quality**: A policy was examined with respect to the number of Core Concepts within it that were rated as 3 or 4 (as either stating a specific policy action to address a Concept or an intention to monitor a Concept) out of the 21 Core Concepts identified; and this ratio was expressed as a rounded up percentage. When several references to a Core Concept were found to be present, the top quality score received was recorded as the final quality scoring for the respective Concept.

(4) Each document was given an **Overall Summary Ranking** in terms of it being of *High*, *Moderate* or *Low* standing according to the following criteria:

(i) *High* = if the policy achieved ≥50% on all of the three scores above.

(ii) *Moderate* = if the policy achieved ≥50% on two of the three scores above.

(iii) *Low* = if the policy achieved <50% on two or three of the three scores above.

### Scoring

Each Core Concept received a score on a continuum from 1 to 4. This was a rating of the quality of commitment to the Core Concept within the policy document:

1 = Concept only mentioned.

2 = Concept mentioned and explained.

3 = Specific policy actions identified to address the concept.

4 = Intention to monitor concept was expressed.

If a Core Concept was not relevant to the document context, it was stated as not applicable.

Each policy document was assessed by two independent raters. For each document, the presence of Core Concepts was assessed for each Vulnerable Group that was identified in the policy. If no Vulnerable Group was mentioned but a Core Concept addressed the total population (e.g. “all people”), the Core Concept was scored as ‘Universal’. The total number and scores for mentioned Core Concepts and Vulnerable Groups was calculated for each document across the three countries.

## Results

**Figure 1:**
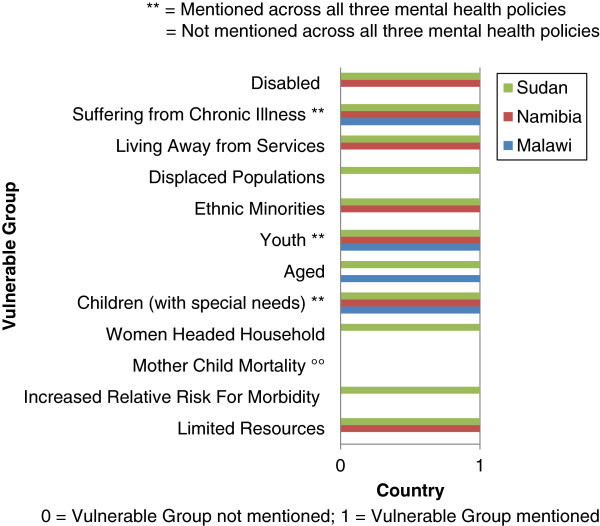
Vulnerable group coverage of the mental health policies of Malawi, Namibia, and Sudan

**Figure 2:**
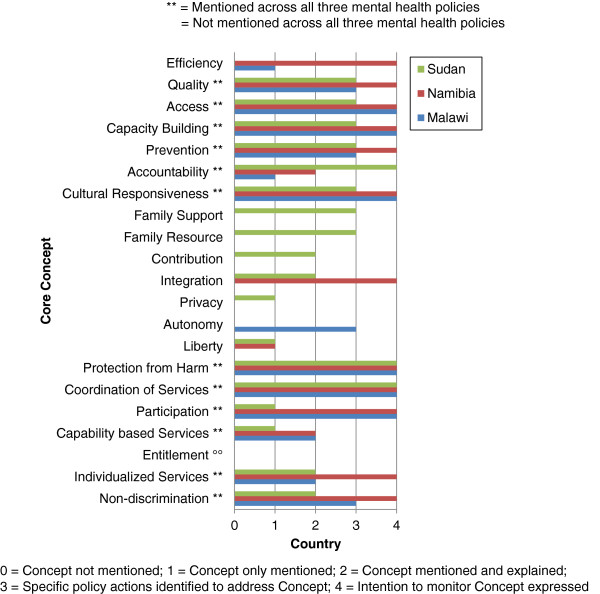
Core concept coverage/core concept quality of the mental health policies of Malawi, Namibia and Sudan.

Table 3 illustrates the results of the mental health policies of Malawi, Namibia, and Sudan across *EquiFrame’s* summary indices. The Vulnerable Group of *Mother child mortality* was not mentioned across the policy documents (Figure 1). Further, in the Malawian and Namibian policies, the Vulnerable Groups of *Increased relative risk for morbidity*, *Women headed household*, and *Displaced populations* were not mentioned. While the Core Concept of *Entitlement* was not mentioned across each of the policies, all three policies exceeded *EquiFrame’s* criterion of 50% on Core Concept Coverage. However, for the Malawian and Namibian policies, the Core Concepts of *Privacy*, *Family resource*, *Family support*, and *Contribution* were not mentioned. For the Namibian and Sudanese policies, the Core Concept of *Autonomy* was not mentioned (Figure 2). Particularly noteworthy was the mental health policy of Sudan, which, while scoring only 48% on Core Concept Quality and therefore receiving an Overall Summary Ranking of *Moderate*, scored 92% on Vulnerable Group Coverage, and 86% on Core Concept Coverage. Having considered more general findings from the application of the framework to these three policies, more detailed findings are now presented with respect to individual country mental health policies.

**Table 3:** ***EquiFrame *summary indices for the mental health policies of Malawi, Namibia, & Sudan**

**Mental health policy**	**VG%**	**CC%**	**Core concept quality%**	**Overall summary ranking**
**Malawi**	33	67	47	*Low*
**Namibia**	58	71	57	*High*
**Sudan**	92	86	48	*Moderate*

### Malawi

The overall goal of the Malawian National Mental Health policy is to provide comprehensive and accessible mental health care services to all citizens of Malawi, in line with the National Health Policy. The policy provides guidance on the following issues: programme development and management; decentralisation and integration into primary, secondary and tertiary health care levels; mental health education; human resources development; mental health care needs of special groups (children and adolescents, the elderly, offenders who are mentally ill, vagrant mentally ill persons and destitutes, victims and perpetrators of violence, aggression, torture, other forms of abuse and disasters, women with special needs, drug and alcohol use/abusers); psychosocial rehabilitation; quality assurance; research; mental health information system; and mental health programme financing.

**Vulnerable Group Coverage** for this policy was 33%. The Vulnerable Group of *Suffering from chronic illness* was mentioned 13 times, *Children with special needs* was mentioned 3 times, while *Aged* and *Youth* were each mentioned once. The remaining Vulnerable Groups, that is, *Limited resources*, *Increased relative risk for morbidity*, *Mother child mortality*, *Women headed households*, *Ethnic minorities*, *Displaced populations*, *Living away from services*, and *Disabled* were not mentioned in the policy. It is notable that the policy actually most frequently used ‘universal’ terminology however, addressing the total population 121 times.

**Core Concept Coverage** for this policy was 67%. A number of Core Concepts were not mentioned in the policy, explicitly, *Privacy*, *Liberty*, *Family resource*, *Family support*, *Integration*, *Contribution*, and *Entitlement*. The most frequently mentioned Core Concept was *Capacity building* (mentioned 74 times), followed by *Coordination of services* (mentioned 24 times), and *Participation* (mentioned 10 times). The remaining Core Concepts were each mentioned less than 10 times, that is, *Prevention* (mentioned 6 times), *Cultural responsiveness*, *Quality*, and *Access* (each mentioned 5 times), *Non-discrimination* (mentioned 4 times), *Capability based services* (mentioned 3 times), *Individualized services* (mentioned 3 times), *Protection from harm* (mentioned twice), and *Autonomy*, *Accountability*, and *Efficiency* (each mentioned once).

With regards to **Core Concept Quality**, ten of the Core Concepts mentioned were scored as 3 or 4. Core Concept Quality was therefore assessed as 47%. The Core Concepts of *Protection from harm*, *Participation*, *Cultural responsiveness*, *Coordination of services*, *Capacity building* and *Access* were each mentioned with an intention to monitor expressed. The Core Concepts of *Prevention*, *Autonomy*, *Non-discrimination*, and *Quality* were each mentioned in relation to specific policy actions to address the Concept. The Core Concepts of *Capability based services* and *Individualized services* were mentioned and explained. The Concepts of *Accountability* and *Efficiency* were each only mentioned in the policy.

While the Malawian National Mental Health Policy scored above 50% for Core Concept Coverage, it scored below 50% for Core Concept Quality and Vulnerable Group Coverage. This policy therefore scored below 50% on two of three of *EquiFrame’s* summary indices, and received an **Overall Summary Ranking** of *Low* (see Table 3).

### Namibia

The goal of the Namibian National Policy for Mental Health is to achieve and maintain a high standard of mental health and well-being in the population of Namibia, and reduce stigma against people with mental disorders. This is to be achieved through the development of a comprehensive community-based mental health service that is decentralized and integrated into the general health service. The policy emphasizes that mental health is a component of the overall health care system. It ensures practical steps to move away from current centralized, curative and hospital based mental health services to one that is comprehensive and integrated into the general health care system based on the Primary Health Care approach and strongly supported by intersectoral collaboration and community participation.

**Vulnerable Group Coverage** for this policy was 58%. The Vulnerable Groups of *Children with special needs* and *Youth* were each mentioned 4 times, while *Suffering from chronic illness* was mentioned twice, and *Limited resources*, *Ethnic minorities*, *Living away from services*, and *Disabled* were each mentioned once. The following Vulnerable Groups were not mentioned in the policy: *Mother child mortality*, *Increased relative risk for morbidity*, *Women headed household*, *Aged*, and *Displaced populations*.

**Core Concept Coverage** for this policy was 71%. Six Core Concepts were not mentioned in the policy: *Autonomy*, *Privacy*, *Family resource*, *Family support*, *Contribution* and *Entitlement*. The Core Concept of *Prevention* was mentioned most frequently (mentioned 6 times), followed by *Protection from harm* (mentioned 4 times), *Integration* (mentioned 4 times), *Non-discrimination*, *Coordination of services*, *Capacity building* (each mentioned 3 times), *Participation*, *Liberty*, *Accountability*, and *Access* (each mentioned twice), and *Cultural responsiveness*, *Capability based services*, *Individualized services*, *Quality*, and *Efficiency* (each mentioned once).

With respect to **Core Concept Quality**, twelve of the Core Concepts mentioned were scored as 4. Core Concept Quality for this policy was therefore 57%. These Core Concepts were: *Protection from harm*, *Prevention*, *Participation*, *Non-discrimination*, *Cultural responsiveness*, *Integration*, *Coordination of services*, *Capacity building*, *Individualized services*, *Quality*, *Access*, and *Efficiency*. The Core Concepts of *Capability based services* and *Accountability* were mentioned and explained. The Core Concept of *Liberty* was only mentioned.

The policy scored above 50% on each of *EquiFrame’s* summary indices of Vulnerable Group Coverage, Core Concept Coverage, and Core Concept Quality. Accordingly, the **Overall Summary Ranking** of the Namibian Mental Health Policy was *High*.

### Sudan

The Sudanese Mental Health Policy comes as a response to the 2001 World Health Assembly’s Ministerial Round Table, the Alma Ata Declarations of Health and the MDGs, while also building on the Sudan national health policy. The main vision of the policy is to provide comprehensive integrated based coverage and quality mental health care to all citizens of Sudan. The document is composed of 27 pages covering three main parts; the first includes the need for a strategy and a situation analysis, the second addresses the policy vision, mission, values and objectives, while the third part covers the policy statement including its principles and action areas. This policy is under endorsement, however it is expected that the final document will be disseminated through the Federal System for implementation at states level. With respect to quality assurance of service delivery and advice of the FMOH, it is expected to be carried out by the newly developed national institute of mental health.

The policy scored 92% with respect to **Vulnerable Group Coverage**. Only the Vulnerable Group of *Mother child mortality* was not mentioned in the policy. The Vulnerable Group of *Disabled* was mentioned most frequently at 33 times. The Vulnerable Groups of *Youth* and *Displaced populations* were each mentioned 4 times. *Increased relative risk for morbidity*, *Children with special needs*, and *Aged* were each mentioned 3 times. *Limited resources* and *Women headed households* were each mentioned twice, while *Ethnic minorities*, *Living away from services*, and *Suffering from chronic illness* were each mentioned once. It is noteworthy that the policy referred to the ‘universal’ population 10 times. On such occasions, no Vulnerable Group was mentioned but a Core Concept addressed the total population.

With regards to **Core Concept Coverage**, 86% of Core Concepts were mentioned in the policy. The policy failed to mention the Core Concepts of *Autonomy*, *Entitlement* and *Efficiency*. The most frequently mentioned Concepts comprised *Protection from harm* (mentioned 11 times), followed by *Prevention* (mentioned 8 times), and *Access* (mentioned 6 times). These were followed in frequency by *Cultural responsiveness* (mentioned 4 times), *Non-discrimination*, *Integration*, *Coordination of services*, *Capacity building*, *Individualized services* and *Quality* (each mentioned 3 times). Finally, *Liberty* and *Family resource* were cited in the policy (each mentioned twice), alongside *Privacy*, *Participation*, *Family support*, *Contribution*, *Capability based services* and *Accountability* (each mentioned once).

Ten of the Core Concepts mentioned were scored as 3 or 4. Accordingly, **Core Concept Quality** was scored as 48%. The Core Concepts of *Protection from harm*, *Coordination of Services* and *Accountability* were each mentioned with an intention to monitor expressed. The Core Concepts of *Prevention*, *Cultural responsiveness*, *Family resource*, *Family support*, *Capacity building*, *Quality*, and *Access* were each mentioned in conjunction with specific policy actions that addressed the Concepts. The Concepts of *Non-discrimination*, *Integration*, *Contribution*, and *Individualized services* were each mentioned and explained. The Concepts of *Privacy*, *Participation*, *Liberty*, and *Capability based services* were each only mentioned.

The policy scored below 50% on *EquiFrame’s* summary index of Core Concept Quality, while scoring above 50% on Vulnerable Group Coverage and Core Concept Coverage. The **Overall Summary Ranking** for the Sudanese Mental Health policy was *Moderate*.

## Discussion

Substantial variability was identified for *EquiFrame’s* summary indices across the mental health policies of Malawi, Namibia, and Sudan. Particular deficits were evident in the Malawian Mental Health Policy in terms of Vulnerable Group Coverage, Core Concept Quality and the resulting Overall Summary Ranking of this policy, but so too the Sudanese policy in terms of Core Concept Quality.

### Vulnerable group coverage

Particularly alarming was the performance of the Malawian Mental health policy with respect to Vulnerable Group Coverage, explicitly mentioning only one-third of vulnerable groups. The Vulnerable Group of *Mother child mortality* was not mentioned across the mental health policy documents however. Further, the Vulnerable Groups of *Increased relative risk for morbidity*, *Women headed household*, and *Displaced populations* were not explicitly mentioned in the Malawian and Namibian mental health policies. Mental health concerns are more prevalent among particular social groups than others, to some degree due to socioeconomic and cultural position in society [10]. Mental health difficulties frequently interplay with other vulnerability factors that may generate susceptibility to double discrimination and multiple disadvantage: *Limited resources*[1,9,12,14,42-44]; *Mother child mortality*[13,14,45]; *Women headed households*[1,10,45]; *Children with special needs*[46-48]; *Aged*[44,49]; *Youth*[1,44,50-52]; *Ethnic minorities*[8,10]; *Displaced populations*[53,54]; *Living away from services*[42,55]; *Suffering from chronic illness*[9,14,43,56]; *Disabled*[57,58]. Formal recognition and incorporation in these mental health policies of specific mechanisms of exclusion and detailed needs of these populations is required to ensure their equitable access to healthcare.

### Core concept coverage/quality

Each of the policies notably exceeded *EquiFrame’s* criterion of 50% on Core Concept Coverage. However, all three of the policies failed to mention the Core Concept of *Entitlement*, relating to the indication by a policy of the way in which vulnerable groups may qualify for specific benefits relevant to them. Disability benefits are necessary for persons with mental disorders at similar rates to those granted to persons with physical disabilities [8]. Further, for the Malawian and Namibian policies, the Core Concepts of *Privacy*, *Family resource*, *Family support*, and *Contribution* were not explicitly mentioned. The Principles for the Protection of Persons with Mental Illness and the Improvement of Mental Health Care [59] stipulates that, ‘Every patient in a mental health facility shall, in particular, have the right to full respect for his or her: (b) privacy’. Regarding the Core Concepts of *Family resource* and *Family support*, as enshrined in the United Nations Convention on the Rights of Persons with Disabilities [7], ‘the family is the natural and fundamental group unit of society and is entitled to protection by society and the State, and that persons with disabilities and their family members should receive the necessary protection and assistance to enable families to contribute towards the full and equal enjoyment of the rights of persons with disabilities’ [38]. With respect to *Contribution*, it is evident that the majority of persons with mental disabilities face disproportionate barriers in obtaining employment; employment and income-generating opportunities must be generated for persons with mental and psychosocial disabilities [43]. Furthermore, for the Namibian and Sudanese policies, the Core Concept of *Autonomy* was not explicitly mentioned. The principle of free and informed consent is the cornerstone of treatment for mental disorders [8]. It is notable that Core Concept Quality was below *EquiFrame’s* criterion of 50% for the Malawian and Sudanese mental health policies. Mechanisms to monitor human rights in mental health facilities are critical, although a limited number of countries have established such mechanisms [60].

### Methodological issues

Both through the process of undertaking this policy analysis initiative and by providing feedback of results to stakeholder workshops in different countries, several factors were observed that are important to consider when interpreting the results of our analysis. Stakeholders including persons with disabilities and their representative organizations during the consultations that took place throughout the development of *EquiFrame* argued that some documents use the term “all”, as in “all people” to be fully inclusive and therefore reference to specific vulnerable groups is not necessary. Indeed, subsidiary analysis of the use of “all”, or its synonyms, indicates that documents using such ‘all-inclusive’ terms, also specify certain vulnerable groups, but not others. Accordingly, it is important to establish which vulnerable groups are included and those that are not, as the use of inclusive terminology does not necessarily address the concerns of specific vulnerable groups [36].

While *EquiFrame* has been developed for the purposes of policy analysis, we believe that this form of analysis can also be usefully applied to other types of planning and guiding documents, and that the coverage of Core Concepts of human rights and the inclusion of Vulnerable Groups is pertinent to a range of diverse guiding documents too. Fuller understanding of the content of any such documents can always be and should always be strengthened by understanding of the context in which the document was developed, the process of its development and the implementation actions that must accompany it for it to take effect. However, describing policy ‘on the books’ is not only a legitimate practice, but a vital one, if we are to recognize and develop documents that are most likely to support human rights and promote greater inclusion in health service provision. If we fail to do this, we risk privileging some groups over others, perhaps addressing the concerns of dominant groups, particularly in the context of services provided through international aid support [11].

Health policy analysis may be beneficial both retrospectively and prospectively, in the understanding of past policy failures and successes and the development of future policy implementation [61]. Accordingly, we believe that the utility of *EquiFrame*, as a policy analysis tool, will extend beyond its application as a framework for evaluation to the development of new policy documents and to the revision of existing documents. By highlighting some high quality health policy documents, *EquiFrame* can navigate those developing policies towards some best-practice examples of human rights coverage and vulnerable group inclusion. It can also provide a check-list of factors for consideration, as well as indicating specific terms and phrasing for use in a policy. It is important to note that since this framework was used to perform what was inherently a content analysis, it is bound by the limitations of using such a methodology, including expertise required to perform this type of analysis. Further, the framework requires the use of two independent raters, generating some scope for diverging interpretations of the material analyzed.

The universality of human rights is contested and it may be argued that interpretations are subject to cultural values and contextual realities [62]. Accordingly, any analysis of human rights or inclusion in health policies is inherently necessarily going to reflect certain cultural and contextual factors. The reflexivity of the analyst – that is, their awareness of their positioning and how this affects their understanding of a policy – is therefore of critical importance. Interpretations do not arise in isolation from who the analysis is performed by, for whom, and in what context. Although these complex issues are very important, it is equally important to recognize that in many instances the pragmatic reality of lived exclusion is hurtful, frequently resulting in needless mortality, and often all too easy to recognize by the failure to address it in health policies.

In order to realize the hope that better policies will be associated with better healthcare, empowerment and inclusion of vulnerable and marginalized groups must occur in the process of policy development and efforts to implement such policies, as well as in policy documents. The practice of power, privilege, and dominance, in local and national policy contexts, and also in the context of programs supported through international aid, will continue to undermine aspirations for equity [11,63]. Without inclusive and effective means of policy development and implementation, policy ‘on the books’ will be inert. Perfectly equitable heath policies will only contribute to inclusion if cognate policies in other sectors embrace similar principles, and if they are translated in measurable actions. While this has not been the focus of this paper, it is necessary for the potential benefits of better written policy to become a reality.

## Conclusions

One of the most crucial steps towards delivering judicious and comprehensive mental health care is the formulation of a policy and plan that will navigate mental health systems and services development [1]. While imperative to improving conditions for people with mental disabilities, mental health policies are absent or deficient in the majority of countries of the world however [2], with forty percent of countries as yet without a dedicated mental health policy [3]. Central to a mental health policy that translates into truly effective and justly distributed health service provision is a health policy fortified by human rights and underpinned by equitable access to health services requiring that priority is afforded to vulnerable groups. Addressing mental health problems in vulnerable groups can support development outcomes more generally, including improved participation in economic, civic, and social activities [9]. Restoration of mental health is not only crucial for individual well-being therefore, but is also critical for economic growth and reduction of poverty in countries [64]. By providing a policy analysis framework of core concepts of human rights and vulnerability, *EquiFrame* may operate as a novel and valuable tool in the evaluation and revision of existing mental health policies, and in the development of the copious mental health policies that as now evident are yet to be formulated. Evaluating, revising, and developing mental health policies through an equity lens that aims to extend health services to the most vulnerable and marginalized and using a broader human rights framework is an important practical and moral initiative in the successful realization of the Millennium Development Goals, and so too the Movement for Global Mental Health, perhaps all-important.

## Competing interests

The authors declare that they have no competing interests.

## Authors’ contributions

**HM**: conception and design, analysis and interpretation of data; drafting the article, revising it critically for important intellectual content. **SET**: conception and design, analysis and interpretation of data; drafting the article, revising it critically for important intellectual content. **MML**: conception and design, analysis and interpretation of data; drafting the article, revising it critically for important intellectual content. **MA**: conception and design, analysis and interpretation of data. **JMV**: drafting the article, revising it critically for important intellectual content. **AM**: conception and design, analysis and interpretation of data. **GVR**: conception and design, analysis and interpretation of data. All authors read and approved the final manuscript.
